# 2376. Trends in COVID-19 vaccination among reproductive-aged in Jamaica: a comparison of two time periods

**DOI:** 10.1093/ofid/ofad500.1997

**Published:** 2023-11-27

**Authors:** Jodian Pinkney, Laura Bogart, Kamali Carroll, Lenroy Bryan, Givana Witter, Dina Ashour, Rocio M Hurtado, Ilona T Goldfarb, Emily P Hyle, Christina Psaros, Bisola Ojikutu

**Affiliations:** Massachusetts General Hospital , Boston, Massachusetts; RAND Corporation, Santa Monica, California; University of the West Indies, Kingston 7, Kingston, Jamaica; University of the West Indies, Kingston 7, Kingston, Jamaica; University of the West Indies, Kingston 7, Kingston, Jamaica; Massachusetts General Hospital, Boston, Massachusetts; Massachusetts General Hospital, Boston, Massachusetts; Massachusetts General Hospital, Boston, Massachusetts; Massachusetts General Hospital, Boston, Massachusetts; Massachusetts General Hospital, Boston, Massachusetts; Massachusetts General Hospital, Boston, Massachusetts

## Abstract

**Background:**

In Jamaica, COVID-19 vaccines have been recommended for pregnant women since August 2021, yet data detailing COVID-19 vaccination behaviors among pregnant Jamaican women have been limited. Specifically, it is unknown if COVID-19 vaccination among pregnant Jamaican women has increased over time, similar to trends observed in the United States.

**Methods:**

A convenience sample of reproductive-aged Jamaican women were recruited from a teaching hospital during two distinct time periods (TP): (1) February 2022 and (2) May-July 2022. Participants self-identified as pregnant (versus nonpregnant) and completed a web-based survey that assessed self-reported COVID-19 vaccination and medical mistrust beliefs. We analyzed the relationship between TP and a predetermined set of variables using chi-square or Fisher’s exact tests. We used logistic regression to estimate adjusted odds ratios (aOR) and 95% confidence intervals (CI) for self-reported COVID-19 vaccination by TP, adjusting for age, education, and comorbidities.

**Results:**

Of the 429 participants, 178 (41%) completed the survey in TP1 and 251 (59%) in TP2 (Table 1). Self-reported data, including trusted sources of COVID-19 vaccination and type of COVID-19 vaccine received, did not differ significantly between TP1 and TP2 (Table 1). Self-reported COVID-19 vaccination among pregnant women was 35% in TP1 vs. 60% in TP2 (aOR (95% CI): 2.78 (1.40 - 5.50). Pregnant women were less likely to agree with the medical mistrust belief, “I don’t trust the COVID-19 vaccine,” in TP2 compared with TP1 (aOR (95% CI): 0.43 (0.18 - 0.99). Self-reported vaccination and medical mistrust beliefs among nonpregnant women did not differ between TP1 and TP2.

**Figure:**
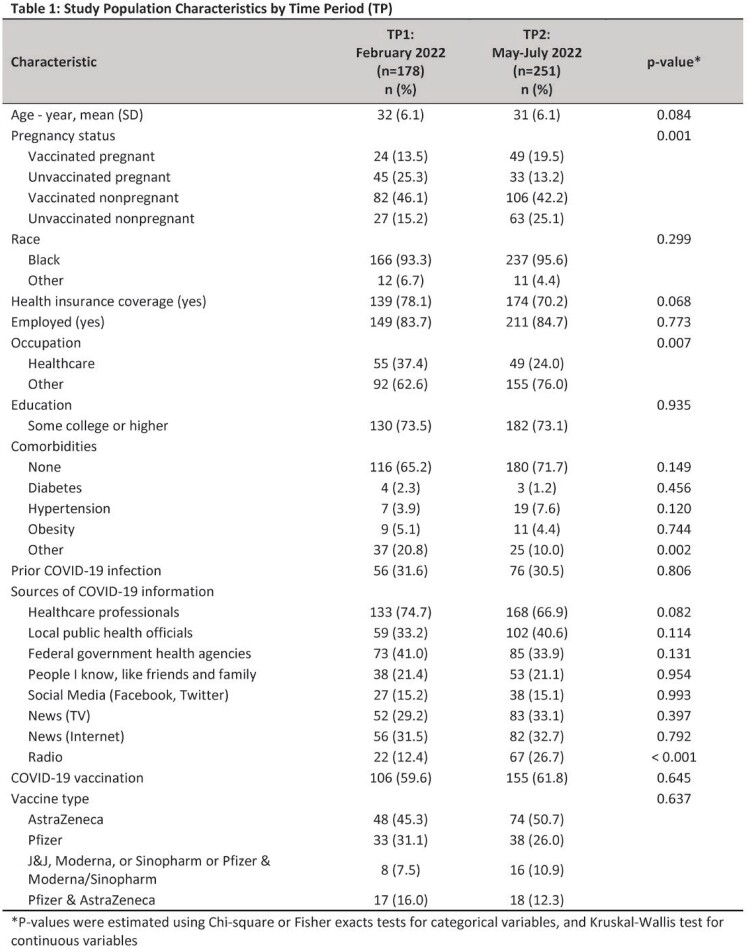

**Figure:**
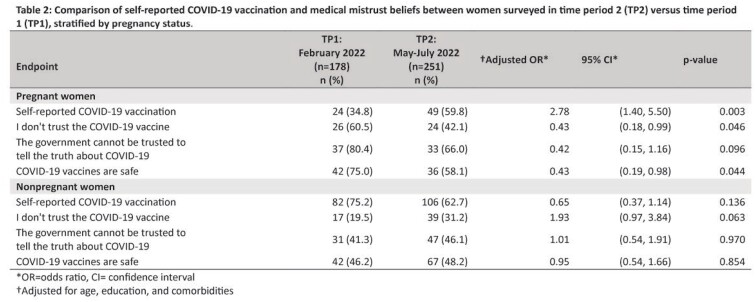

**Conclusion:**

Self-reported COVID-19 vaccination among pregnant Jamaican women was significantly higher in TP2 compared with TP1, while medical mistrust related to COVID-19 vaccination was lower. To improve the efficacy of vaccination campaigns and enhance the communication of vaccine safety between pregnant women and their healthcare providers in the future, it is crucial to identify trends in COVID-19 vaccination and medical mistrust beliefs among pregnant women over time.

**Disclosures:**

**Emily P. Hyle, MD MSc**, UpToDate.com: Royalties

